# Efficacy and safety of Chinese herbal medicine Wen Xin granules for the treatment of unstable angina pectoris with Yang deficiency and blood stasis syndrome: study protocol for a randomized controlled trial

**DOI:** 10.1186/s13063-021-05771-y

**Published:** 2021-11-13

**Authors:** Pan-pan Tian, Qing-juan Wu, Jun Li, Heng-wen Chen, Ji Wu, Ya-wen Deng, Zi-cong Xie, Wei Zhao, Yu-qing Tan

**Affiliations:** 1 Guang’anmen Hospital, China Academy of Chinese Medical Sciences, Beijing, China; 2 Graduate School Department, Beijing University of Chinese Medicine, Beijing, China

**Keywords:** Coronary heart disease, Unstable angina pectoris, Wen Xin decoction, Chinese herb medicine, Randomized controlled trial

## Abstract

**Introduction:**

Unstable angina pectoris (UAP) is the common type of coronary heart disease with the risk of developing into acute myocardial infarction (AMI). Currently, there are still numerous patients suffering from recurrent angina after revascularization or conventional medication due to the microvascular lesions, endothelial dysfunction, chronic inflammation, in-stent restenosis, and other factors. As an important part of China’s medical and health care system, traditional Chinese medicine (TCM) has rich clinical experience in the treatment of UAP. According to the theory of TCM, Yang deficiency and blood stasis syndrome is a common type of UAP. Wen Xin decoction, as a type of Chinese herbal medicine, has been used in the clinic for years and shown great efficacy in the treatment of UAP with Yang deficiency and blood stasis syndrome. This study aims to evaluate the efficacy and safety of Wen Xin granular in patients with UAP.

**Methods and analysis:**

This is a double-blinded, randomized, placebo-controlled clinical trial. A total of 502 participants will be randomly allocated to the intervention group and the placebo group. Based on conventional medication, the intervention group will be treated with Wen Xin granular and the placebo group will be treated with Wen Xin granular placebo. The primary outcomes are major adverse cardiovascular events (MACE). Assessments will be performed 1 year after the treatment. The secondary outcomes include TCM symptom scale score, Seattle angina questionnaire, and thromboelastography. Assessments will be performed at baseline (before randomization) and 4 and 8 weeks after randomization.

**Discussion:**

This trial will provide high-quality data on the benefits and risks of Wen Xin granular in patients with UAP.

**Trial registration:**

ClinicalTrials.govNCT04661709. Registered on 30 November 2020

## Introduction

Coronary heart disease (CHD), aka ischemic heart disease, has remained as the leading cause of death worldwide over the past decade (WHO 2018) [1]. As one of the risk types of CHD, unstable angina pectoris (UAP) is a clinical syndrome intermediate in severity between stable angina and acute myocardial infarction (AMI). It is also referred to as crescendo or pre-infarction angina [2]. UAP is thought to be caused by a progression in the severity and extent of coronary atherosclerosis, coronary artery spasm, or bleeding into non-occlusive plaques in the coronary artery. It eventually can result in complete occlusion of the artery [3]. The medication therapy for UAP mainly includes antiplatelet, anticoagulation, vasodilation, and relieving coronary constriction. Revascularization including coronary artery bypass grafting (CABG) and percutaneous coronary intervention (PCI) is indicated effective in certain high-risk individuals and also has been shown to improve angina. However, even after revascularization, a substantial percentage of patients return with recurrent or continued angina, requiring newer and better therapies [4–6]. Standard drug therapy and invasive revascularization are effective in decreasing progression to infarction, reducing symptoms and multiple hospitalizations, but without a decrease in the long-term mortality rate in most cases [7]. There are still many patients suffering from persistent or recurrent angina after standard medical therapy and/or revascularization [8, 9]. The causes may be related to coronary spasm, thrombosis, in-stent restenosis, untreated vascular stenosis, coronary microvascular lesions, or psychological factors [10]. Chinese herbal medicine (CHM) either in single herb or in herbal formula has been commonly used for treating UAP in China for many years. And more and more researches are focusing on the exploration of effective Chinese herbal formula for the prevention and treatment of UAP. According to the theory of traditional Chinese medicine (TCM), all the related symptoms and signs in a certain disease phase are generalized to a syndrome (*Zheng* in Chinese), a basic unit and key concept of TCM [11]. Based on years of clinical experience and previous researches, we identified that *Yang* deficiency and blood stasis syndrome is the main pathogenesis of UAP. In TCM theory, *Yang* and *Yin* are the most fundamental concepts, which represent two inter-opposite aspects existing within one object or the two inter-opposite things or forces. *Yang* is generally associated with items or concepts that are warm, bright, moving, and ascending [12]. *Yang* has the function of promoting blood circulation. If *Yang* is deficient and unable to promote blood circulation, the blood stasis syndrome will be formed. Therefore, we consider ‘Warming *Yang* and activating blood circulation’ as the main principle of treatment. As an experienced prescription, Professor Li Jun has used Wen Xin decoction for the treatment of UAP with *Yang* deficiency and blood stasis syndrome in the clinic for about 20 years. Previous studies showed that Wen Xin decoction could regulate blood lipid, improve hemodynamics and the clinical symptoms, and reduce inflammation and the incidence of non-fatal myocardial infarction [13–16]. However, due to the small sample size and the relatively short follow-up period, the curative effect of Wen Xin decoction still needs to be verified by large-scale clinical trials. This multi-center, double-blind, randomized, placebo-controlled trial aims to evaluate the efficacy and safety of Wen Xin granules in patients with UAP.

## Methods/design

### Trial organization

Three clinical researchers will be trained to master the standards and methods of case collection to minimize selection bias. In the process of data collection, the patients’ medication situation and the treatment situation outside the plan will be recorded in detail to exclude interference and contaminating factors in the statistical analysis. An independent data and safety monitoring board has been established and will monitor the conduct and safety of this trial. Stopping guidelines and monitoring practices have been established. The trial is sponsored by the State Administration of Traditional Chinese Medicine (China) (ref: JDZX2015249). The funders have no role in the study design, data collection and analysis, preparation of the manuscript, or decision to publish.

### Study population

A total of 502 patients will be recruited from two centers: Guang’anmen Hospital, the China Academy of Chinese Medical Sciences, and Beijing Anzhen Hospital Affiliated to Capital Medical University.

### Recruitment of participants

Two strategies are used to recruit patients with UAP. One is to display recruitment posters outside the clinics at the same time publicize posters through WeChat. The posters contain a brief introduction about the population required, the Chinese herbal medicine treatment offered to eligible participants, and the contact information of the researchers. The other is to recruit participants in outpatient clinics from the Department of Cardiovascular medicine of Guang’anmen Hospital, the China Academy of Chinese Medical Sciences, and Beijing Anzhen Hospital Affiliated to Capital Medical University. Three researchers (P-P T, Q-J W, Y-W D) take part in enrolling participants, and another two researchers (Z-C X, W Z) are responsible for assigning participants to interventions. Patients who meet the study criteria and want to join this study will be requested to sign a written informed consent form. The consent form includes the nature, objectives, potential benefits, and consequences of the study. The personal information of participants will be kept private, and only authorized study staff will have access to this information. All the paper forms about this study will be kept in a locked, secure office. In addition, the consent form details the required supportive care, the name of the principal investigator responsible for the protocol, and the patient’s right to accept or refuse treatment and to terminate participation and withdraw from the study.

### Patient and public involvement

Patients were involved in the design of this study. During the feasibility stage, the priority of the research question, the selection of outcome measures, and the methods of recruitment were informed by discussion with patients through a focus group. During the study, a patient joined the independent trial steering committee. Cardiovascular specialists from Anzhen Hospital and Guang’anmen Hospital also identified this research as an area of priority for the clinicians treating patients with CHD. Once the trial has been published, participants will be informed of the results by email. During this study, free tests and Chinese herbal medicine Wen Xin granular will be provided for patients.

### Inclusion criteria

Participants are included if they are aged between 35 and 75 years; diagnosed with CHD through coronary arteriography; clinically diagnosed with UAP in low or medium risk, and belonging to ‘Yang deficiency and blood stasis syndrome’ according to TCM; and give written informed consent. For the diagnostic criteria of UAP, we will refer to “2014 AHA/ACC Guidelines for the Diagnosis and Management of Non-ST-Elevation Acute Coronary Syndromes” [17]. For the TCM diagnostic criteria, we will refer to “Guidelines for Clinical Research into New Traditional Chinese Medicine Drugs for Chest Obstruction” [18] (2002 edition).

### Exclusion criteria

Criteria for exclusion are chest pain caused by congenital heart diseases, valvular heart disease, severe neurosis, or arrhythmia, with New York Heart Association class III or IV heart failure, in the acute phase of cerebral infarction, with uncontrolled hypertension (systolic blood pressure >160 mmHg and/or diastolic blood pressure >95 mmHg in the resting state), with uncontrolled hyperglycemia or diabetic complications, with mental and neurological abnormalities or dysgnosia, female patients in pregnancy or lactation, or by participating in other clinical trials.

### Handling of withdrawal and data management

Participants will be free to withdraw from this study at any time upon request. The investigator may terminate a participant from the study if the participant meets a newly developed or not previously recognized exclusion criterion that precludes further participation or a participant stops taking the study medicine or takes other medicine which may affect the accuracy of the results before the end of this study. Patients lost to follow-up and patients who withdraw from this study will be recorded and reported. The data collected in this trial will comprise information recorded on case report forms. If less than 5–10% of the participants withdraw from the study, then incomplete data will be cleaned. If the rate is larger than 10%, the missing data will be processed through data imputation, intention-to-treat analysis, and sensitivity analysis. The data will be entered by trial investigators using the double-entry method.

### Interventions

All participants will go through an interview to know more information about this study. After obtaining informed consent from each participant and completing a baseline evaluation, patients who meet the inclusion criteria and none of the exclusion criteria are randomly divided into the intervention group or the placebo group. Both groups will receive conventional medication treatment, including antiplatelet, lipid-lowering, coronary-dilating, antihypertension, and hypoglycemic drugs. The specific drugs include aspirin 100 mg qd, clopidogrel bisulfate 75 mg qd, atorvastatin 20 mg qn, isosorbide mononitrate tablets 20 mg bid, tartaric acid metoprolol 12.5–25 mg bid, and trimetazidine dihydrochloride tablets 20 mg tid. Besides, the intervention group will receive Wen Xin granules and the control group receive placebo granules for 8 weeks. TCM granules are compound preparations of Chinese herbs, the main components of which are shown in Table 1. Placebo granules are composed by 5% of Wen Xin granular, starch, dextrin, and artificial pigment. The placebo granules are similar to TCM granules in the aspects of color, smell, taste, and package. Participants are required not to take other Chinese herbal decoctions or Chinese patent medicine during the treatment.

### Randomization and blinding

**Fig. 1:**
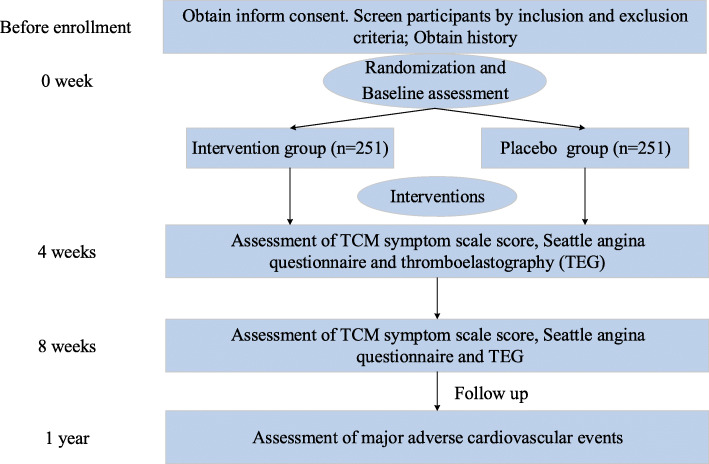
The flow chart of the randomized clinical trial of Wen Xin granules for the treatment of unstable angina pectoris

Randomization is performed by an independent statistician (JW). The randomization sequence was generated by using SAS software (version 9.4). All drugs provided by the pharmacy department will be numbered with a label in accordance with the randomization schedule. This trial is a double-blind trial and includes two levels of blinding. The first level is for the group corresponding to the interventions (the intervention and placebo groups), and the second level is for case number corresponding to the groups (group A and group B). The numbers are kept in opaque sealed envelopes. The two levels of blinding will be sealed separately and given to the leader of the clinical research. Emergency letters will be sent to the participating centers, saved with the test drug, and properly preserved until the end of the trial. Treatment assignments will not be revealed to the patients and investigators (including statisticians) until the entire study has been completed. The time points are shown in Fig. 1.

**Table 1:** Main components of Chinese herbal medicine Wen Xin granules

Chinese name	Latin name	English name	Medicinal part	Dosage/(g)
**Dang Shen**	Salviae Miltiorrhizae Radix et Rhizoma	*Dan-Shen* root	Tuber root	15
**Rou Gui**	Cinnamomi Cassiae Cortex	Cassia bark	Dry bark	3
**Jiang Huang**	Curcumae Longae Rhizoma	Turmeric	Tuber root	9
**Xi Xin**	Asari Radix et Rhizoma	Manchurian wildginger	Rhizome	3
**Mai Dong**	Ophiopogonis Radix	Dwarf lilyturf tuber	Tuber root	12
**Chi Shao**	Paeoniae Radix Rubra	Red paeony root	Rhizome	15
**Chuan Xiong**	Ligusticum chuanxiong Hort	*Sichuan* Lovase rhizome	Rhizome	10
**E Zhu**	Curcumae Rhizoma	Acruginous turmeric rhizome	Tuber root	9
**Quan Xie**	Scorpio	Scorpion	Dry body	9
**Shan Zha**	Crataegi Fructus	Crataegus Pinnatifida	Fruit	15
**Gua Lou**	Trichosanthis Fructus	Mongolian snakegourd fruit	Fruit	10
**Da Huang**	Rhei Radix et Rhizoma	Rhubarb root and rhizome	Rhizome	6
**Zhi Gan Cao**	Glycyrrhizae Radix et Rhizoma	Liquorice root	Rhizome	6

### Primary outcomes

The primary outcome measure is the incidence of 1-year risk of all-cause mortality and major adverse cardiovascular events (MACE) after the intervention. The MACE is defined as the composite endpoint of acute myocardial infarction, heart failure, cardiac revascularization (PCI or CABG), ischemic stroke, and cardiovascular mortality [19].

### Secondary outcomes

**Fig. 2:**
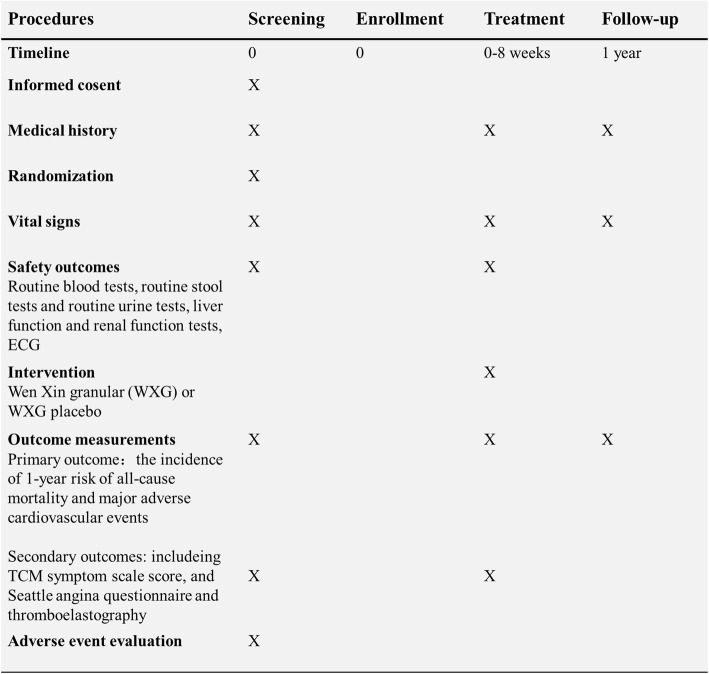
SPIRIT figure

The secondary outcomes include TCM symptom scale score and Seattle angina questionnaire (SAQ) and thromboelastography (TEG). The TCM symptom scale score includes 7 items which are shown in Table 2. The SAQ regroups 19 items measuring five specific scales: physical limitations, anginal stability, anginal frequency, treatment satisfaction, and disease perception targeting a specific disease and treatment group. Parameters assessed by TEG are R (represent clotting time), K and Angle (reflect clot strength and development), MA (maximum platelet-fibrin clot strength), CI (represents overall coagulability), and LY30 (represents lysis). Assessments are performed at baseline, 4 weeks, and 8 weeks after intervention (Fig. 2).

**Table 2:** Traditional Chinese medicine symptom scale of Yang deficiency and blood stasis syndrome

Traditional Chinese medicine symptoms		Score
Chest pain	None	□0
	Mild (chest pain attacks at least 2–3 times a week or 1–3 times a day, but the pain is not serious and lasts several minutes each time. Sometimes nitroglycerin is needed)	□2
	Moderate (chest pain attacks several times every day, and is more serious, may last several minutes to about 10 min each time. Nitroglycerin is generally needed)	□4
	Severe (chest pain attacks several times every day and lasts for a long time, which affect the activities of daily life. Nitroglycerin is always needed)	□6
Chest tightness	None	□0
	Mild (slight chest tightness)	□2
	Moderate (obvious chest tightness with sighing respiration sometimes)	□4
	Severe (chest tightness like suffocation with sighing respiration always)	□6
Aversion to cold and cold limbs	None	□0
	Mild (slight cold limbs)	□1
	Moderate (cold limbs, need to put on more clothes to keep warm)	□2
	Severe (aversion to cold and cannot be relived through putting on more clothes)	□3
Palpitation	None	□0
	Mild (feel slight discomfort sometimes)	□1
	Moderate (feel obvious discomfort. Palpitation attacks sometimes ang lasts for a long time)	□2
	Severe (palpitation often happens and even affects life)	□3
Shortness of breath	None	□0
	Mild (shortness of breath after general activity)	□1
	Moderate (feel shortness of breath after a little bit activity)	□2
	Severe (feel shortness of breath even without physical activity)	□3
Slightly dark tongue	No	□0
	Yes	□1
Deep and thin pulse	No	□0
	Yes	□1
Total score

### Safety outcomes

The safety outcomes include the following: (1) measurement of heart rate, blood pressure, temperature, and respiration; (2) routine blood tests, routine stool tests, and routine urine tests; (3) liver function tests (alanine aminotransferase, aspartate aminotransferase, γ-gamma glutamyl transferase, alkaline phosphatase, and total bilirubin) and renal function tests (blood urea nitrogen and creatinine); (4) electrocardiogram (mainly ST-T changes); and (5) records of adverse events at any time. These biological indicators will be monitored from the grouping of these patients until the end of the study.

### Adverse events

Some adverse reactions, such as mild nausea, stomach distention, and heart rate decrease, may occur after medication. All these reactions and other unexpected symptoms are recorded as adverse events. The start date, end date, degree, relationship to the trial medicine, and whether the patients withdraw from the study will be recorded. Severe adverse events are required to be reported to the lead researcher of the trial, ethics committees, and sponsors within 24 h, and the participants will be provided with any necessary treatment. If the adverse event persists, follow-up will continue until the adverse event has resolved.

### Sample size

The incidence of MACE will be compared between the two groups. The rate of MACE, as suggested in a previous study, is expected to be 12.7% in the intervention group and 24.5% in the control group [15]. The following formula for a two-group trial will be used [20]:

$$ n=\frac{{\left({U}_{\alpha }+{U}_{\beta}\right)}^22P\left(1-P\right)}{{\left({P}_1-{P}_0\right)}^2} $$

Based on *α* = 0.05 and *β* = 0.1, the required sample size per group is approximately 228 participants. Allowing for 10% attrition, we will aim to recruit 502 participants, with 251 patients in each group. Thus, we will recruit a total of 242 patients for this trial.

### Data analysis

Data analysis will be conducted by statisticians who are independent from our research team. The study’s statistical and analytical plan has been decided in advance. All data analysis will be conducted using SPSS 22.0 software. Student’s test will be performed on continuous normally distributed variables, the Wilcoxon rank sum test on non-normal variables, and Pearson’s *χ*^2^ test on categorical variables. Data are presented as means ± standard deviation (‾x±*s*) for continuous variables or percentage values for categorical variables. The statistical significance level will be set at *p* < 0.05 and all the statistical tests were two-sided [21].

### Frequency and plans for auditing trial conduct

We do not specify a formal auditing plan. However, rigor and fidelity to the approved study protocol are monitored through weekly meetings and monthly meetings. Weekly meetings of staff engaged in enrollment and data collection, and monthly meetings of the full study team.

## Discussion

Syndrome differentiation and treatment is a central focus of TCM. According to the theory of TCM, UAP can be divided into a variety of syndromes according to different clinical manifestations. Among these syndromes, Yang deficiency and blood stasis syndrome is the most common type in middle-aged and elderly patients who generally manifest as chest tightness, chest pain, aversion to cold, lack of warmth in the limbs, shortness of breath, tiredness, palpitation, and loose stool. Wen Xin decoction is a compound prescription of TCM which contains 13 Chinese herbs. Most of the herbs have been proved to have cardiovascular pharmacological effects. For instance, the extracts of *Codonopsis pilosula* can protect myocardial cells, enhance hematopoietic function, inhibit platelet aggregation, and improve heart function [22]. *Pericarpium trichosanthis* extract, Rhizoma Chuanxiong extract and tanshinone, Danshensu, catecholamine, and salvianolic acid A in *Salvia miltiorrhiza* have effects of protecting myocardium, anticoagulant, antiplatelet aggregation, antiatherosclerosis, and anti-inflammatory [23–25]. Although previous studies have identified that Wen Xin decoction was effective in improving clinical symptoms of patients with UAP. However, its long-term effect still needs further observation. Besides, the sample sizes of those studies were small and no studies reported the method of random sequence generation or described allocation concealment. Blinding is an essential method for preventing research outcomes from being influenced by either the placebo effect or the observer bias. However, no study reported the blinding of participants. Moreover, no placebo control was used to mask researchers and participants. This double-blind, randomized, placebo-controlled trial was carried out to test the short-term and long-term effects of Wen Xin granular through the comparison between an intervention group and a placebo group. To ensure appropriate high-quality methodology and strict quality control, this protocol was developed in accordance with the Standard Protocol Items: Recommendations for Interventional Trials 2013 [26]. The method of recruitment, randomization and allocation concealment, and data collection are described in detail.

Our study has some limitations. One limitation is that decoctions were used in previous studies, but granules are applied in this study. Because placebo decoction is more difficult to make than granular placebo. In another way, compared with decoctions, granules are more convenient for patients, and patient compliance may be increased. Another limitation is that the intervention time is relatively short since the prescription always needs to be adjusted according to the change of patients’ symptoms.

## Data Availability

None declared.

## Abbreviations

UAP: Unstable angina pectoris
AMI: Acute myocardial infarction
TCM: Traditional Chinese medicine
MACE: Major adverse cardiovascular events
CHD: Coronary heart disease
CABG: Coronary artery bypass grafting
PCI: Percutaneous coronary intervention
CHM: Chinese herbal medicine
SAQ: Seattle angina questionnaire
TEG: Thromboelastography
